# Supplementary material for: Context-specific regulation of surface and soluble IL7R expression by an autoimmune risk allele
Source: Nat Commun. 2019 Oct 8;10:4575. doi: 10.1038/s41467-019-12393-1 (PMC6783569; doi:10.1038/s41467-019-12393-1)
Supplement: Supplementary file 1 — Supplementary Information [file 41467_2019_12393_MOESM1_ESM.pdf]

Context-specific regulation of monocyte surface IL7R expression and soluble receptor secretion by a common autoimmune risk allele

Al-Mossawi et al

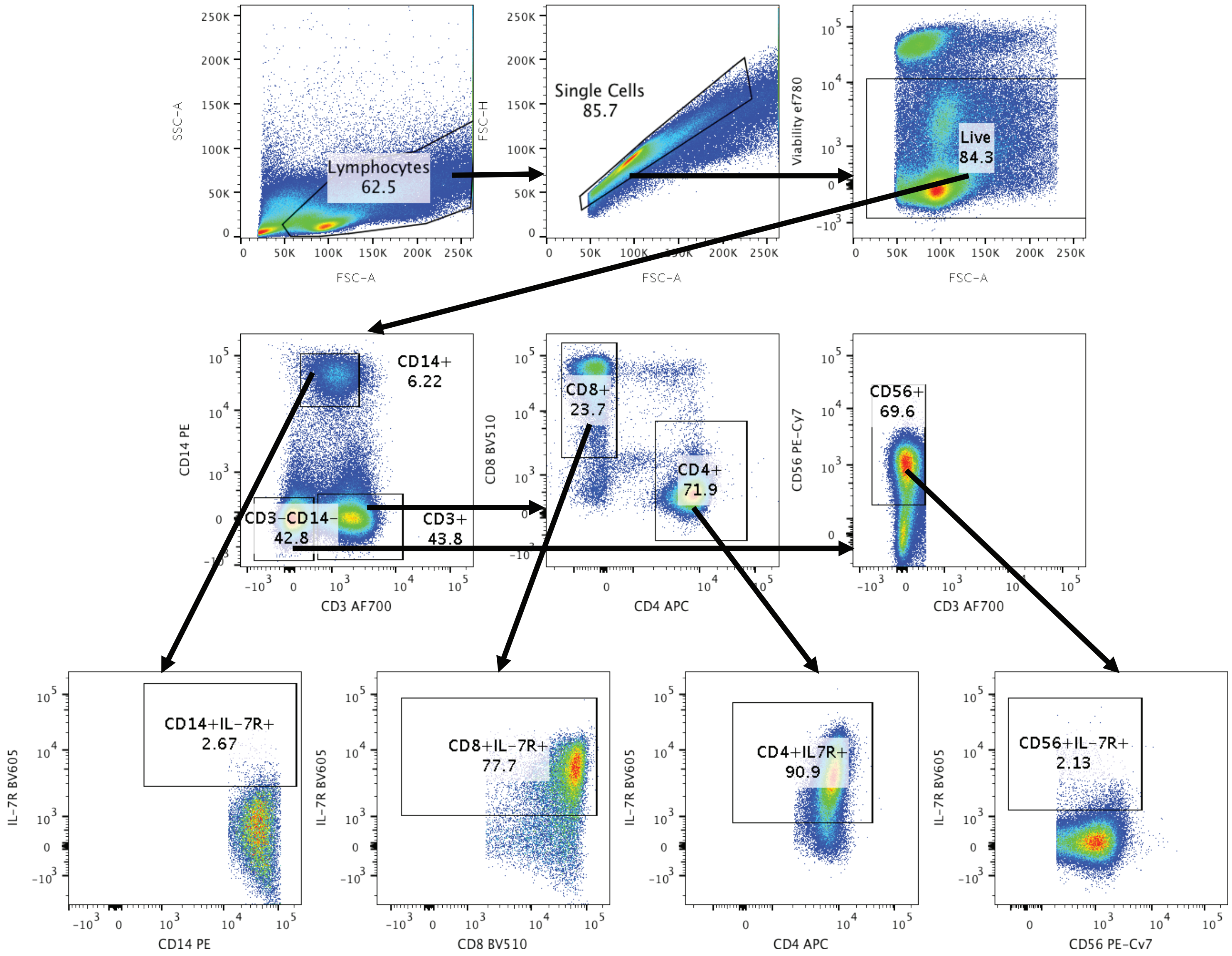

Supplementary Figure 1

Gating strategy for determining cell populations assessed across the cohort - here shown are unstimulated PBMCs illustrating the relative absence of IL7R + monocytes

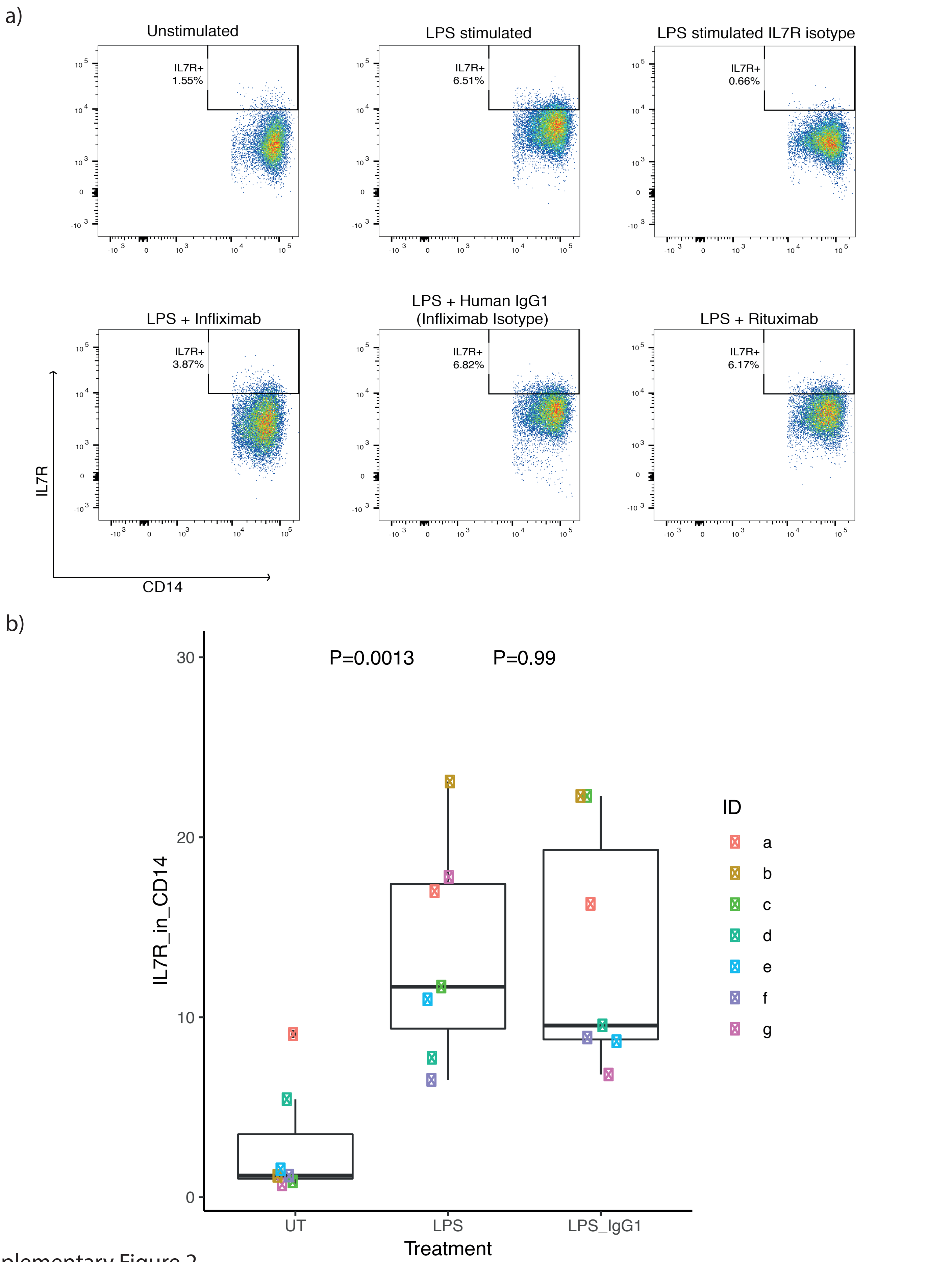

Supplementary Figure 2

a) Representative flow cytometry plots showing IL7R expression on CD14+ cells after treatment with LPS, LPS + Infliximab, LPS + IgG1, and LPS + Rituximab. Gating on CD14+ cells as shown in supplementary figure 1.
b) Data showing IL7R expression on CD14 monocytes before and after treatment with LPS or LPS in the presence of IgG1 isotype antibody at 5ug/ml.

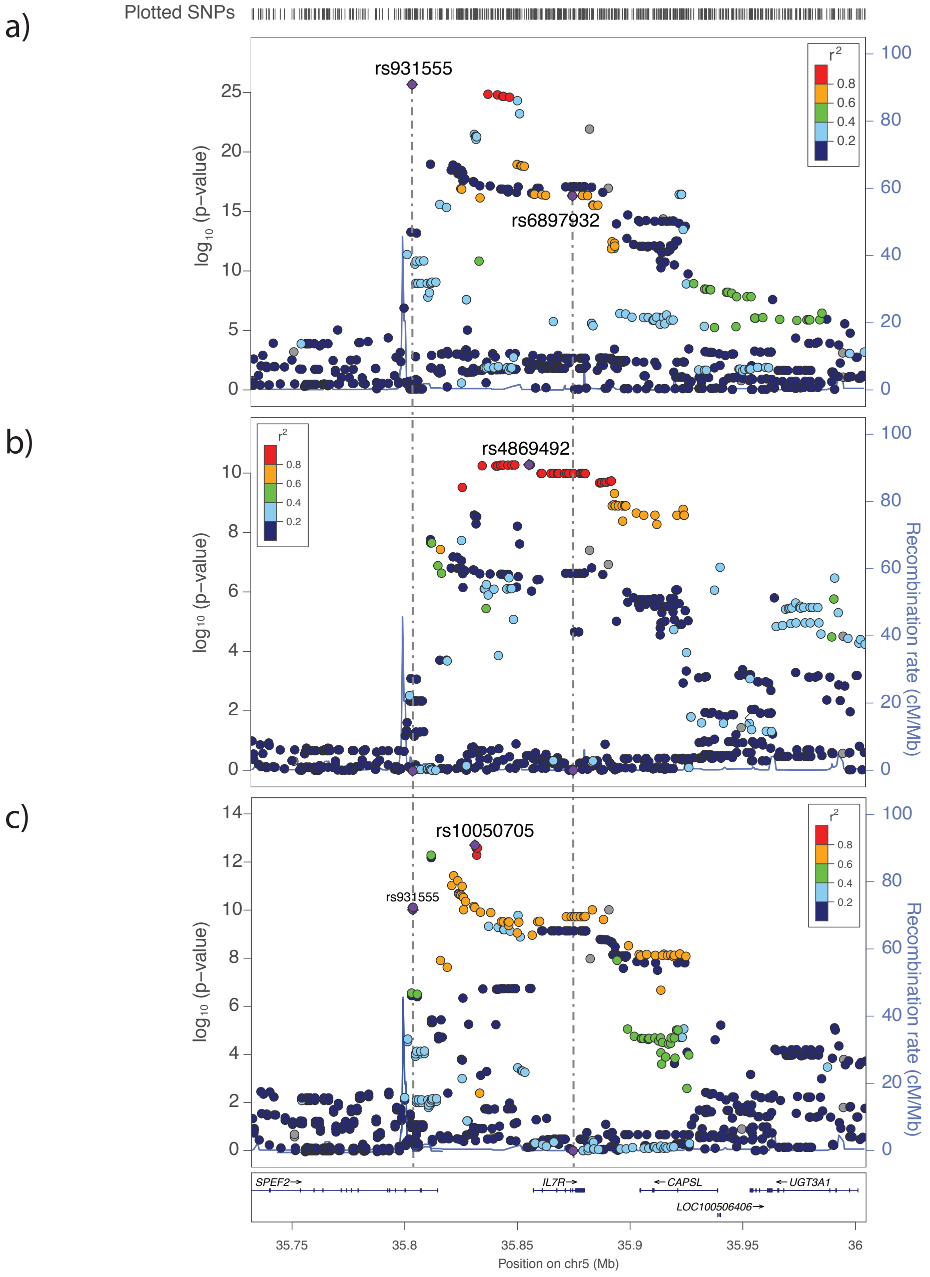

Supplementary Figure 3

a) Peak eQTL for IL7R after LPS maps to rs931555, b) controlling for rs931555 resolves association at rs6897932, c) controlling for rs6897932 carriage leaves residual association at rs931555

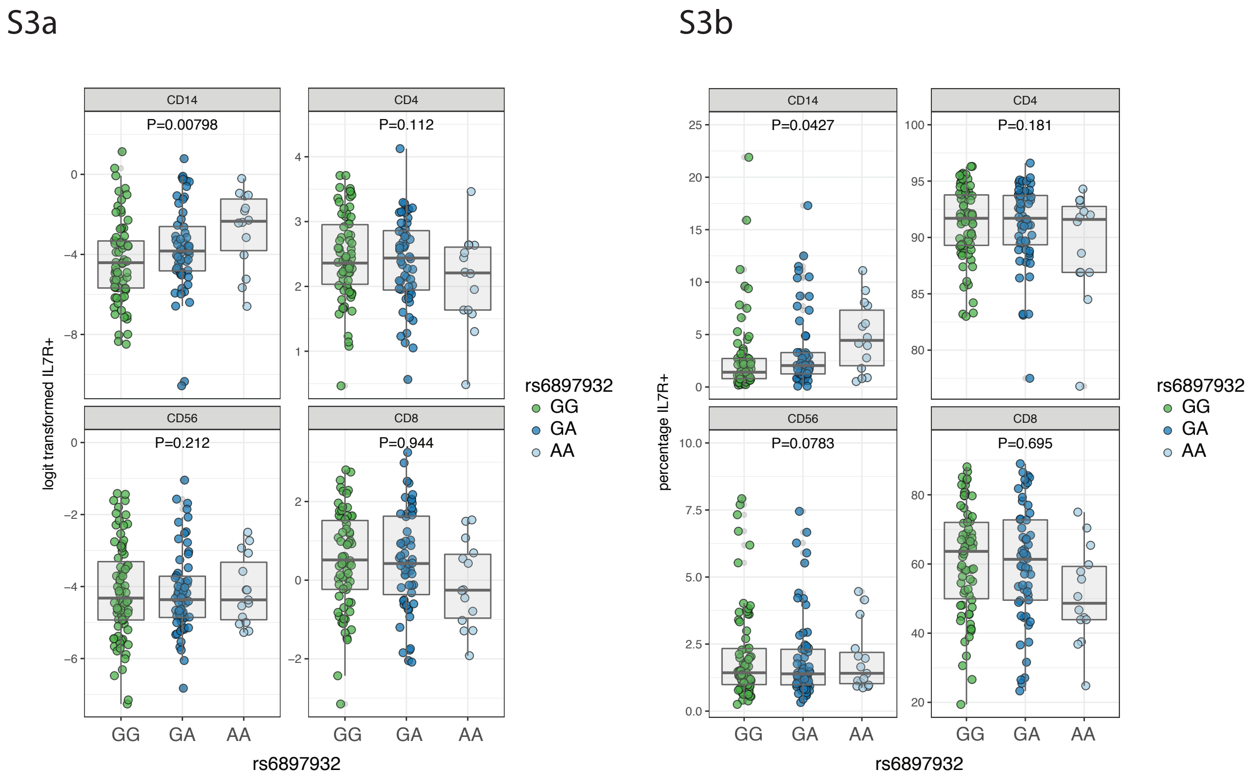

Supplementary Figure 4

Baseline untreated surface IL7R staining by genotype from PBMC cultures a) batch corrected log values, b) raw data; both showing significant effect of rs68979342 carriage on monocytes surface IL7R levels

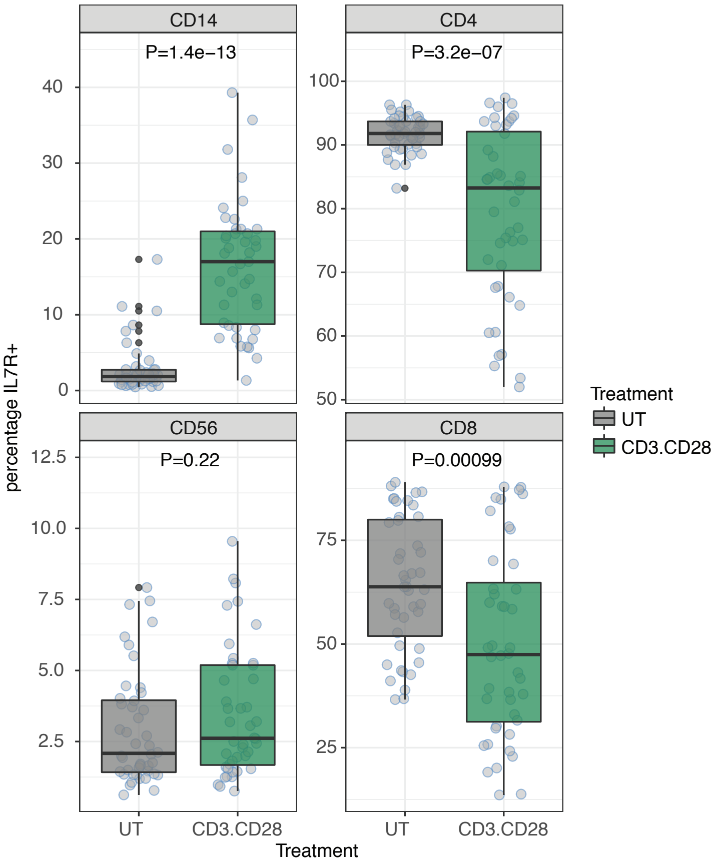

Supplementary Figure 5

PBMC cultures were treated with CD3.CD38 beads for 24h and IL7R staining measured with flow cytometry. Significant induction of CD14 IL7R$^+$ was noted upon T-cell specific stimulation indicating cross-talk between cell types. Experiments performed in n=44 subjects over 4 batches. A genetic effect of rs6897932 was not observed in any cell type (P>0.05).

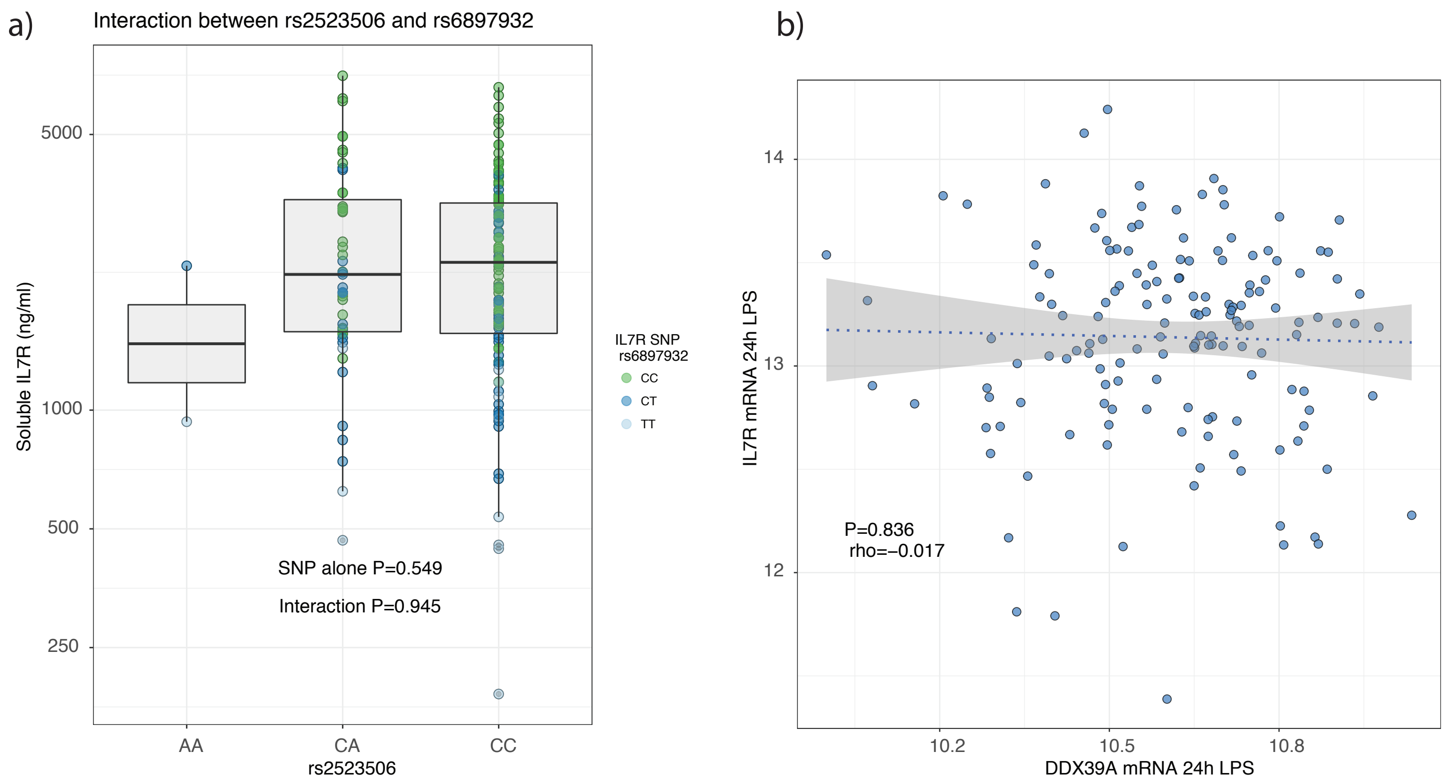

Supplementary Figure 6

a) No significant effect of rs2523506 on monocyte derived sIL7R was detected.
A significant interaction between rs2523506 and rs6897932 was not observed
b) There was no relationship between DDX39A expression and IL7R expression

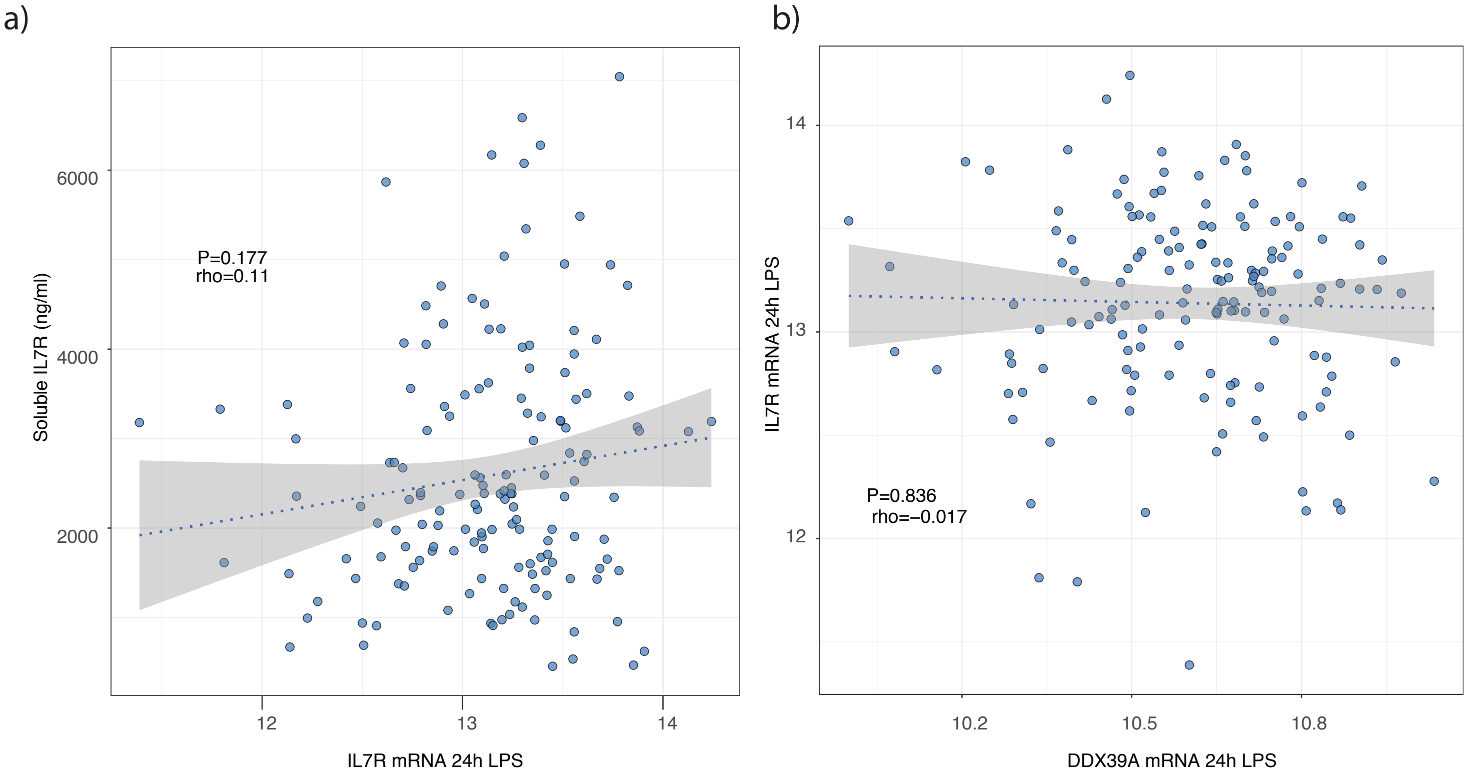

Supplementary Figure 7

a) No significant relationship between total RNA measured (probe to pan transcript 3′ UTR) and soluble IL7R demonstrating predominate post-translational control of sIL7R
b) No significant relationship between DDX39A expression and IL7R expression

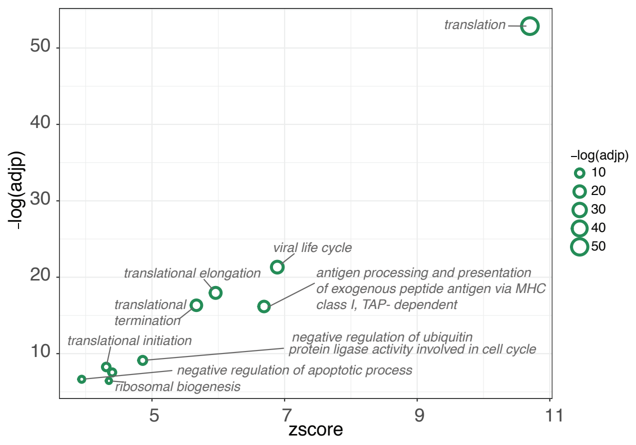

Supplementary Figure 8

Results from gene ontology biological pathway analysis was performed on IL-7 induced genes with all significant pathways labelled.

a)

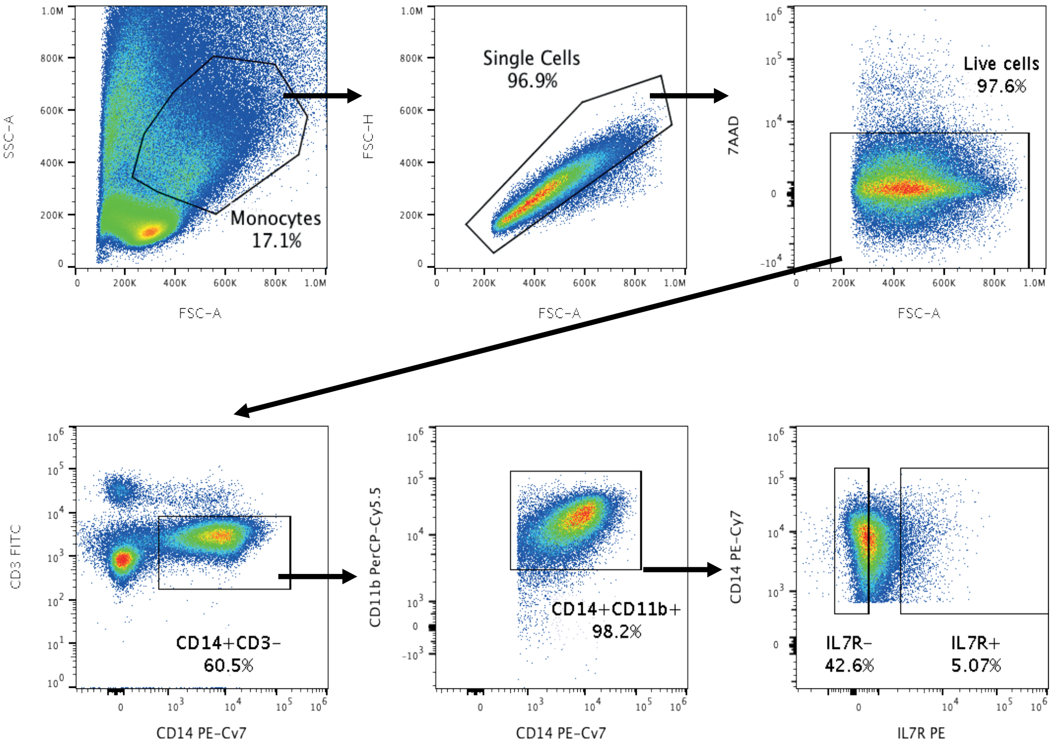

b)

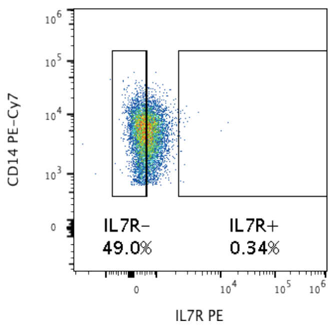

c)

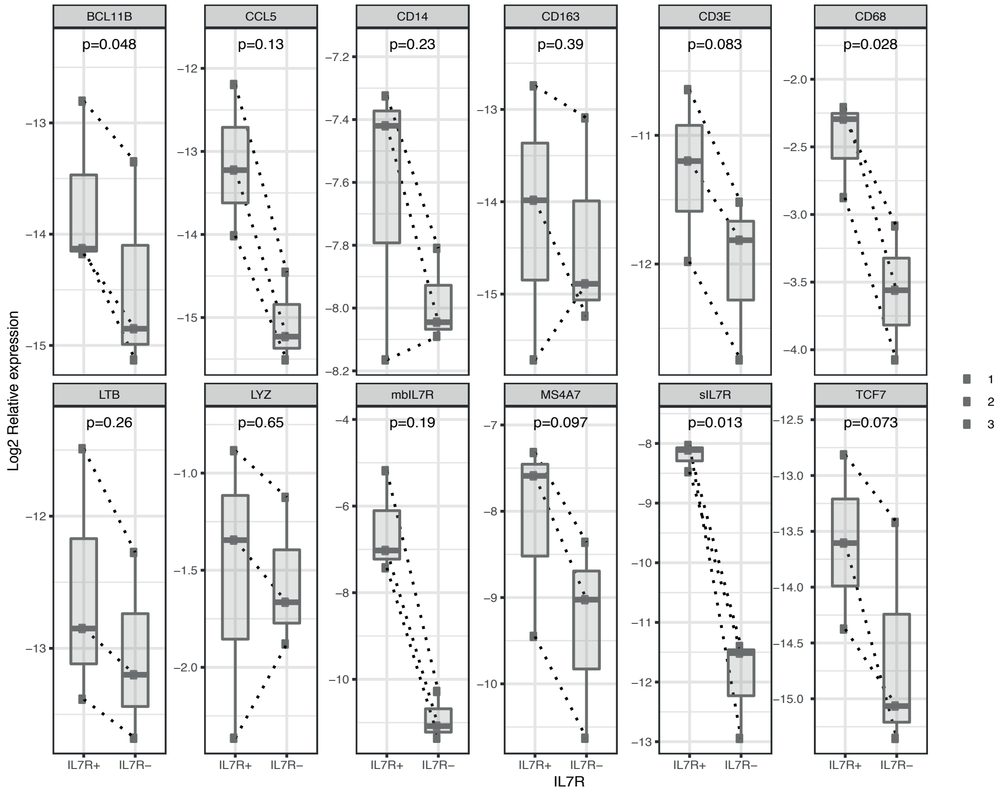

Supplementary Figure 9
a) Sorting strategy for spoindyloarthritis synovial monocytes

b) IL7R isotype control used for setting IL7R+ sorting gate

c) Expression of CD14, CD163, LYZ, MS4A7, FCGR3A, membrane bound -IL7R (mbIL7R) and soluble IL7R (sIL7R) by qPCR from sorted synovial IL7R+ and IL7R- CD14+CD11b+ monocytes, n = 3, paired t-test.

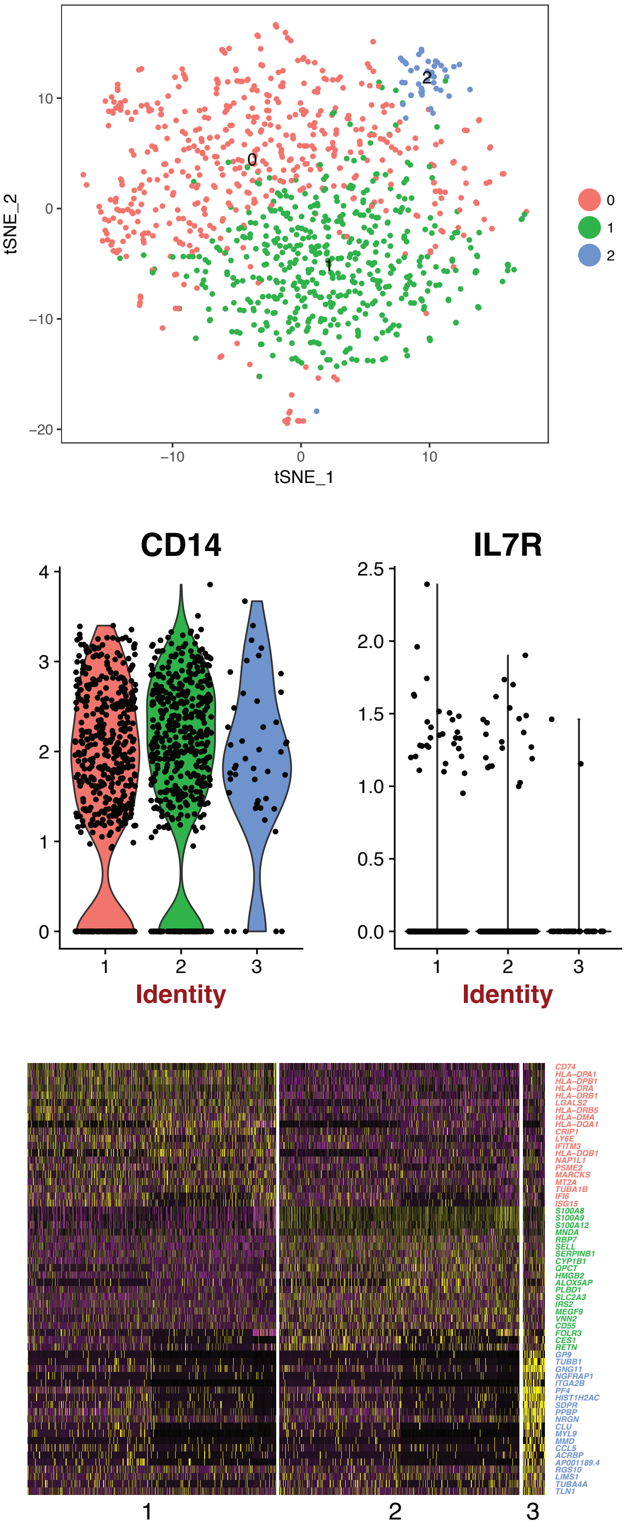

Supplementary Figure 10

a) t-SNE clustering of monocytes from single-cell RNA sequencing of spondyloarthritis patient PBMC (n=3).
b) Violin plots showing CD14 and IL7R expression in each of the three identified clusters.
c) Heatmap of top 20 genes from each of the clusters identified in the spondyloarthritis PBMC monocytes.

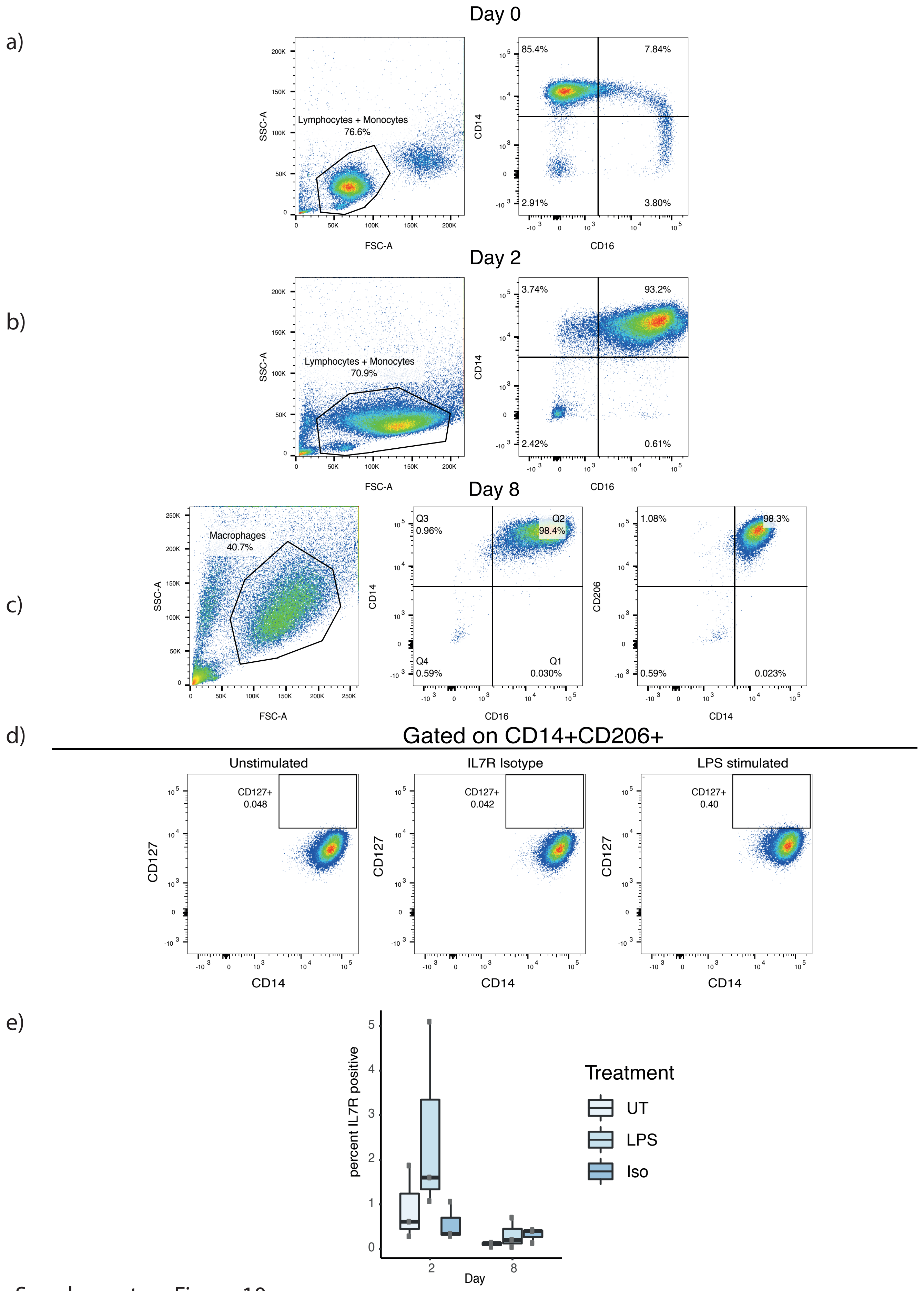

Supplementary Figure 10

a) Positively sorted monocytes from PBMC, day 0
b) Representative CD14 and CD16 expression after 2 days of culture with 50ng/ml of M-CSF
c) Representative CD14 and CD206 expression after 8 days of culture with 50ng/ml of M-CSF
d) Representative IL7R expression on CD206+ Macrophages at day 8 with and without re-stimulation with 20ng/ml of LPS for 24 hours
e) Summary data of IL7R expression on CD206+ macrophages after 8 days of culture with M-CSF.

Supplementary Table 1: Anitbodies used in flow cytometry experiments

Antibody Target	Fluorochrome	Clone	Supplier	Catalogue	Dilution
CD3	AF700	UCHT1	BioLegend	300423	1:50
CD4	FITC	RPA-T4	BioLegend	300506	1:50
CD8α	BV510	RPA-T8	BioLegend	301047	1:50
CD14	PE	TÜK4	Miltenyi	130-113-147	1:100
CD19	PerCP/Cy5.5	SJ25C1	BioLegend	363013	1:50
CD56	PE/Cy7	5.1H11	BioLegend	362509	1:50
CD127 (IL-7Rα)	BV605	A019D5	BioLegend	351333	1:100
IgG1 isotype	BV605	MOPC-21	Biolegend	400161	1:100
Fixable viability dye	eFluor780	N/A	eBiosciences	L34976	1:250

Supplementary Table 2: Primer Sequences

Primers for bulk sorted synovial monocyte qPCR

Target	Forward primer	Reverse primer
**ACTB**	CATGTACGTTGCTATCCAGGC	CTCCTTAATGTCACGCACGAT
**BCL11B**	CGAGAGGAGCTAAGCGCGCGG	GGTGACACGGAGGCAAGTCAGG
**CCL5**	CAGTCGTCCACAGGTCAAGG	CTTGTTCAGCCGGGAGTCAT
**CD14**	GGGATATAAGAGGCAGCCGAA	CGCAGCGGAAATCTTCATCG
**CD163**	TAAAGCCCTGTCTCCTTCCAG	ACTGGTTTCCTGAGCAGATTACA
**CD3E**	GCAGGCAAAGGGGACAAAAC	GCAGTGTTCTCCAGAGGGTC
**CD68**	TCAGCTTTGGATTCATGCAG	AGGTGGACAGCTGGTGAAAG
**LTB**	GAGGACTGGTAACGGAGACGG	GAAACGCCTGTTCCTTCGTC
**LYZ**	CTGTTACGGTCCAGGGCAAG	AACACATCCAGTTTGCTAGGC
**mbIL7R (exons 5-6)**	TCCAACCGGCAGCAATGTAT	GATCCATCTCCCCTGAGCTA
**MS4A7**	TCCCCAGAGGTGAGCTGAT	CAGGATCTGGACAGTCCCAAG
**sIL7R (exons 5-7)**	AGCTCCAACCGGCAGCAATGT	GGCCATACGATAGGCTTAATCCTGAGC
**TCF7**	GACCGCAACCTGAAGACACA	TGTGGTGGATTCTTGGTGCTT

TaqMan primers for single-cell qPCR (all human with FAM-MGB dye)

Target	Assay ID
**IL7Rmb (exons 6-7)**	Hs00904815_m1
**CLEC4E**	Hs00372017_m1
**TNF**	Hs00174128_m1
**CUX1**	Hs00738851_m1
**AKT1**	Hs00178289_m1
**STAB1**	Hs01109068_m1
**CD163**	Hs00174705_m1
**CSF1R**	Hs00911250_m1
**CCL5**	Hs00982282_m1
**DDX39A**	Hs01124952_g1
**DDX39B**	Hs01122168_g1
**MALAT1**	Hs00273907_s1
**LTB**	Hs00242739_m1
**IFNB1**	Hs01077958_s1
**CD14**	Hs00169122_g1
**FCGR3B**	Hs04334165_m1
**LYZ**	Hs00426232_m1
**ACTB**	Hs01060665_g1

TaqMan custom primer for single-cell qPCR

Name	Sequence	Probe position
**sIL7R (exons 5-7)**	TGACACTCCTGCAGAGAAAGCTC<br>CAACCGGCAGCAATGTATGAGAT<br>TAAAGTTCGATCCATCCCTGATCA<br>CTATTTTAAAGGCTTCTGGAGTG<br>AATGGAGTCCAAGTTATTACTTC<br>AGAACTCCAGAGATCAATAATAG<br>CTCAGGATTAAGCCTATCGTATG<br>GCCCAGTCTCCCCGATCATAAGA<br>AGACTCTGGAACATCTTTGTAAG<br>AAACCAAGAAAA	135
