# Supplementary material for: Context-specific regulation of surface and soluble IL7R expression by an autoimmune risk allele
Source: Nat Commun. 2019 Oct 8;10:4575. doi: 10.1038/s41467-019-12393-1 (PMC6783569; doi:10.1038/s41467-019-12393-1)
Supplement: Supplementary file 3 — Reporting Summary [file 41467_2019_12393_MOESM3_ESM.pdf]

nature research

Corresponding author(s): Benjamin Fairfax

Reporting Summary

Nature Research wishes to improve the reproducibility of the work that we publish. This form provides structure for consistency and transparency in reporting. For further information on Nature Research policies, see Authors & Referees and the Editorial Policy Checklist.

Statistical parameters

When statistical analyses are reported, confirm that the following items are present in the relevant location (e.g. figure legend, table legend, main text, or Methods section).

n/a	Confirmed	
☐	☒	The exact sample size (*n*) for each experimental group/condition, given as a discrete number and unit of measurement
☐	☒	An indication of whether measurements were taken from distinct samples or whether the same sample was measured repeatedly
☐	☒	The statistical test(s) used AND whether they are one- or two-sided<br>*Only common tests should be described solely by name; describe more complex techniques in the Methods section.*
☐	☒	A description of all covariates tested
☐	☒	A description of any assumptions or corrections, such as tests of normality and adjustment for multiple comparisons
☐	☒	A full description of the statistics including central tendency (e.g. means) or other basic estimates (e.g. regression coefficient) AND variation (e.g. standard deviation) or associated estimates of uncertainty (e.g. confidence intervals)
☐	☒	For null hypothesis testing, the test statistic (e.g. *F*, *t*, *r*) with confidence intervals, effect sizes, degrees of freedom and *P* value noted<br>*Give P values as exact values whenever suitable.*
☒	☐	For Bayesian analysis, information on the choice of priors and Markov chain Monte Carlo settings
☒	☐	For hierarchical and complex designs, identification of the appropriate level for tests and full reporting of outcomes
☒	☐	Estimates of effect sizes (e.g. Cohen's *d*, Pearson's *r*), indicating how they were calculated
☐	☒	Clearly defined error bars<br>*State explicitly what error bars represent (e.g. SD, SE, CI)*

Our web collection on statistics for biologists may be useful.

Software and code

Policy information about availability of computer code

Data collection	*Provide a description of all commercial, open source and custom code used to collect the data in this study, specifying the version used OR state that no software was used.*
Data analysis	R statistical suite version 3.4.3 "kite-eating tree", R packages: Deseq2, ggplot2, XGR. RNA sequences were mapped with HISAT and counts retrieved with HTseq-count. As listed in methods.

For manuscripts utilizing custom algorithms or software that are central to the research but not yet described in published literature, software must be made available to editors/reviewers upon request. We strongly encourage code deposition in a community repository (e.g. GitHub). See the Nature Research guidelines for submitting code & software for further information.

Data

Policy information about availability of data

All manuscripts must include a data availability statement. This statement should provide the following information, where applicable:

- Accession codes, unique identifiers, or web links for publicly available datasets
- A list of figures that have associated raw data
- A description of any restrictions on data availability

The raw sequencing data generated for the present study has been deposited in the European Bioinformatics ArrayExpress Archive under the following accession codes: E-MTAB-8225, E-MTAB-8207. All raw data used for generating figures has been deposited in the Source Data file.

Field-specific reporting

Please select the best fit for your research. If you are not sure, read the appropriate sections before making your selection.

☒ Life sciences ☐ Behavioural & social sciences ☐ Ecological, evolutionary & environmental sciences

For a reference copy of the document with all sections, see nature.com/authors/policies/ReportingSummary-flat.pdf

Life sciences study design

All studies must disclose on these points even when the disclosure is negative.

Sample size	Empirical data from previous eQTL analysis demonstrated strong eQTL at this locus in cohort of 322 individuals. The effect sizes were such that a cohort of ~150 would be reasonably powered.
Data exclusions	On the few occasions where samples were excluded it was when there was clear failure of flow cytometry antibodies/ reagents. There was no knowledge of genotypes at this point.
Replication	Analysis was performed in parallel of PBMCs and purified monocytes from volunteers - thus one sample acted as a technical replication. The paper was based upon replicating a previous eQTL.
Randomization	All samples were randomly chosen from healthy volunteers
Blinding	Investigators were blinded to genotype until completion of initial flow-cytometry experiments.

Reporting for specific materials, systems and methods

Materials & experimental systems

n/a	Involved in the study	
☒	☐	Unique biological materials
☐	☒	Antibodies
☒	☐	Eukaryotic cell lines
☒	☐	Palaeontology
☒	☐	Animals and other organisms
☐	☒	Human research participants

Methods

n/a	Involved in the study	
☒	☐	ChIP-seq
☐	☒	Flow cytometry
☒	☐	MRI-based neuroimaging

Antibodies

Antibodies used	Listed as: Antigen|Flurophore|Clone|Manufacturer|Cat. No.|Dilution. CD3, AF700, Clone: UCHT, Biolegend: 300423, used at 1:50; CD4, FITC, Clone:RPA-T4, Biolegend:300506, 1:50; CD8a, BV5120, RPA=T8, Biolegend:301047, 1:50; CD14, PE, TUK4, Miltenyi:130-113-147, 1:100; CD19, PerCP/Cy5.5, SJ25C1, Biolegend:363013, 1:50; CD56, PE/Cy7, 5.1H11, Biolegend:362509, 1:50; CD127, BV605, A019D56, Biolegend:351333, 1:100; IgG1, BV605, MOPC-21, Biolegend:400161, 1:100; Fixable viability dye, eFluor780, Biolegend:L34976, 1:250
Validation	As per manufacturers. Bead controls used for flow.

Human research participants

Policy information about studies involving human research participants

Population characteristics	Healthy European volunteers recruited via the Oxford Biobank, age 30:60
Recruitment	recruited via the Oxford Biobank - an NIHR supported biobank for healthy indivduals in the Oxfordshire area

Flow Cytometry

Plots

Confirm that:

☒ The axis labels state the marker and fluorochrome used (e.g. CD4-FITC).

☒ The axis scales are clearly visible. Include numbers along axes only for bottom left plot of group (a 'group' is an analysis of identical markers).

☒ All plots are contour plots with outliers or pseudocolor plots.

☒ A numerical value for number of cells or percentage (with statistics) is provided.

Methodology

Sample preparation	Staining antibodies and dye clones, dilutions and manufacturer shown in Supplementary Table 1. Cells were stained in phosphate buffered saline containing 1% fetal calf serum on ice and in the dark for 20 minutes, then fixed in 1.6% paraformaldehyde. All samples included fixable amine reactive viability dye and isotype control for IL-7R.
Instrument	Flow cytometry was performed on a BD Fortessa calibrated daily with calibration and tracking beads from BD Biosciences.
Software	Data was analysed using FlowJo software (Treestar®).
Cell population abundance	samples were pbmcs and all proportions are listed in the results
Gating strategy	As per gating strategy figure

☒ Tick this box to confirm that a figure exemplifying the gating strategy is provided in the Supplementary Information.
