# Supplementary material for: Context-specific regulation of surface and soluble IL7R expression by an autoimmune risk allele
Source: Nat Commun. 2019 Oct 8;10:4575. doi: 10.1038/s41467-019-12393-1 (PMC6783569; doi:10.1038/s41467-019-12393-1)
Supplement: Supplementary file 4 — Description of Additional Supplementary Files [file 41467_2019_12393_MOESM4_ESM.pdf]

Description of Additional Supplementary Files

Supplementary Data 1: 2hr expression correlates of 24h IL7R expression

Supplementary Data 2: 24hr expression correlates of sIL7R

Supplementary Data 3: IL-7 regulated monocyte transcripts

Supplementary Data 4: Single cell sequencing marker genes for IL7R positive monocyte subset
